# Increased Circulating H3 Histone in Response to Repeated Bouts of Exercise Does Not Associate with Parallel Alterations of Cell-Free DNA

**DOI:** 10.3390/biology10030181

**Published:** 2021-03-02

**Authors:** Robert Stawski, Konrad Walczak, Ewelina Perdas, Anna Prymont-Przymińska, Anna Zwolińska, Piotr Kosielski, Tomasz Budlewski, Gianluca Padula, Hanna Jerczynska, Dariusz Nowak

**Affiliations:** 1 Department of Clinical Physiology, Medical University of Lodz, 92-215 Lodz, Poland; ewelina.perdas@umed.lodz.pl (E.P.); anna.przyminska@umed.lodz.pl (A.P.-P.); 2 Department of Internal Medicine and Nephrodiabetology, Medical University of Lodz, 92-215 Lodz, Poland; konrad.walczak@skwam.lodz.pl; 3 Cell-to-Cell Communication Department, Medical University of Lodz, 92-215 Lodz, Poland; anna.zwolinska@umed.lodz.pl; 4 Academic Laboratory of Movement and Human Physical Performance, Medical University of Lodz, 92-215 Lodz, Poland; piotr.kosielski@umed.lodz.pl (P.K.); gianluca.padula@umed.lodz.pl (G.P.); 5 Department of Rheumatology, Medical University of Lodz, University Hospital Name of the Military Medical Academy-Central Hospital Veterans of Lodz, ul. Pieniny 30, 92-115 Lodz, Poland; tomasz.budlewski@gmail.com; 6 Central Scientific Laboratory, Medical University, Mazowiecka 6/8, 92-215 Lodz, Poland; hanna.jerczynska@umed.lodz.pl

**Keywords:** exercise, histone h3, cell-free DNA, aggregation, interleukins, PAD4

## Abstract

**Simple Summary:**

Histones are also a common disease marker. After PAD4 mediated hypercitrullination extracellular H3Cit exhibits high toxicity contributing to tissue damage which, in cases of systemic inflammation, may lead to multiorgan failure and finally to death. We tested whether circulating histones rise in response to strenuous exercise. Herein, we have observed that circulating histones and PAD4 raised in response to exercise. Despite the parallel increase, no significant correlation between citrullinated histone and aggregation or cell-free nDNA was found. However, positive correlations of cf nDNA with aggregation and PAD4, lactate with aggregation, and lactate with citrullinated histone have been observed.

**Abstract:**

Numerous studies have shown that cf nDNA significantly rises in stress caused by exercise. However, during nuclear decondensation, released DNA is followed by histones. Histones are also a common disease marker. After PAD4 mediated hypercitrullination extracellular H3Cit exhibits high toxicity contributing to tissue damage which, in cases of systemic inflammation, may lead to multiorgan failure and finally to death. We tested whether circulating histones rise in response to strenuous exercise. Eleven average-trained men performed three treadmill exercise tests to exhaustion at speed corresponding to 70% VO2max separated by 72 h of resting. Blood was collected before and just after each bout of exercise and plasma proteins were measured using enzyme-linked immunosorbent assay, whereas platelet activity was estimated with Light Transmission Aggregometry. Both, circulating histones and PAD4 raised in response to exercise. Plasma citrullinated histones increased from 3.1 ng/mL to 5.96 ng/mL (*p* = 0.0059), from 3.65 ng/mL to 6.37 ng/mL (*p* = 0.02), and from 3.86 ng/mL to 4.75 ng/mL (*p* = 0.033) after the first, second, and third treadmill run, respectively. However despite the parallel increase, no significant correlation between citrullinated histone and aggregation or cell-free nDNA was found. Furthermore, positive correlations of cf nDNA with aggregation and PAD4, lactate with aggregation, and lactate with citrullinated histone have been observed.

## 1. Introduction

Histones which together with DNA form nucleosomes, the basic units of chromatin, can be found in circulating plasma both in healthy subjects and in those with some diseases. Extracellular histones released mainly due to cell death have been implicated in the pathogenesis of various serious diseases such as trauma-induced multiple organ dysfunction, sepsis, autoimmune diseases, ischemic heart disease, or even as markers of severity in COVID-19 infection [1,2,3,4,5]. Histones and nuclear DNA (nDNA) might be released from the cellular nucleus separately, simultaneously as nucleosomes or as a component of neutrophil extracellular traps (NETs) when internal structures are released to bind and kill invading microbes [6]. This process is accompanied by a rise in a variety of circulating cytokines including IL-6 and IL-10 [7]. Modification of histone H3 by citrullination is catalyzed by the enzyme peptidylarginine deiminase 4 (PAD4). Weakened binding of citrullinated histone H3 (H3Cit) to negatively charged DNA leads to chromatin decondensation and PAD4/CitH3 dependent NETs formation [8]. Thus, H3Cit is recognized as an in vitro and in vivo marker of NETosis [9,10]. Apart from pathological processes, increased NETosis was described as an integral part of body’s response to vigorous physical exercise [6]. Some studies have proved that cell-free nuclear DNA (cf nDNA) rises in response to various types of exercises [11,12,13,14]. The exercise-induced increase in cf nDNA was many times higher than changes in other biomarkers of metabolic adaptation and muscle damage. Hence, the plasma concentration of cf nDNA seems to be an efficient marker of exercise load and its persistent elevation could be associated with the increased risk of occurrence of overtraining syndrome in athletes [15]. In our previous studies, we found that three repeated bouts of exhaustive treadmill exercise caused the increase in cf nDNA without development of tolerance and decreased the integrity of post-exercise cf nDNA correlated with increased post-exercise whole blood chemiluminescence [11,16]. Because nDNA is wrapped around an octamer of (two H2A, two H2B, two H3, and two H4) histones, it may be expected that exercise would result in an increase in plasma histones concentrations. This hypothesis is supported by the observation of exercise-induced increment of another nuclear protein HMGB1 in humans [12,17]. Extracellular histones can act by the direct interaction with the phospholipid bilayer proceeded by the loss of endothelial membrane barrier function [18], whereas endothelial cells are crucial regulators of vascular hemostasis, and play a pivotal role in the mechanism underlying the development of vascular disorders [19]. Extracellular histones are also able to interact with platelets indirectly, through TLR receptors. Moreover, in vitro-generated NETs induced distinct aggregation of washed human platelets, while excess and prolonged interaction of NETs with platelets in vivo can cause severe inflammation and host organ damage [20,21]. Pharmacological inhibition of histone release, their neutralization in the circulation or inhibition of histone-evoked signal transduction reduced significantly mortality in an animal model of multiple organ injury [22]. To the best of our knowledge, none of the previous studies has investigated the effect of exercise on the concentration of extracellular histones in humans plasma. Therefore, in this study, we aimed to evaluate changes in the extracellular H3Cit, and PAD4 in response to three bouts of exhaustive treadmill run separated by seventy two hours resting period. Furthermore, the associations between plasma histone concentration and cf nDNA, platelet activity, selected cytokines, and markers of metabolic response to exercise were analyzed. In this study we would like to verify how selected factors behave in response to exercise and thus also confirm or deny the harmful impact of circulating histones.

## 2. Materials and Methods

### 2.1. Studied Group

The studied group involved eleven, non-smoking healthy men. All their characteristics as well as the inclusion/exclusion criteria were the same as in our previous articles [11,16]. Briefly, all volunteers who met inclusion criteria were members of the Medical University of Lodz, mean age was 34.0 ± 5.2 years, mean body weight 87.4 ± 13.8 kg, mean body mass index 26.2 ± 3.1 kg/m^2^, maximal oxygen consumption 49.6 ± 4.5 mL/kg*min, forced vital capacity (FVC) 6.09 ± 0.41 L, 106.4 ± 6.4% of predicted, forced exhaled volume in the first second (FEV_1_) 4.93 ± 0.45 L, FEV1/FVC 80.9 ± 5.6%. All volunteers provided a written informed consent. The protocol was reviewed and approved by the Ethics Committee of the Medical University of Lodz (RNN/95/14/KB).

### 2.2. The Study Design

**Figure 1:**
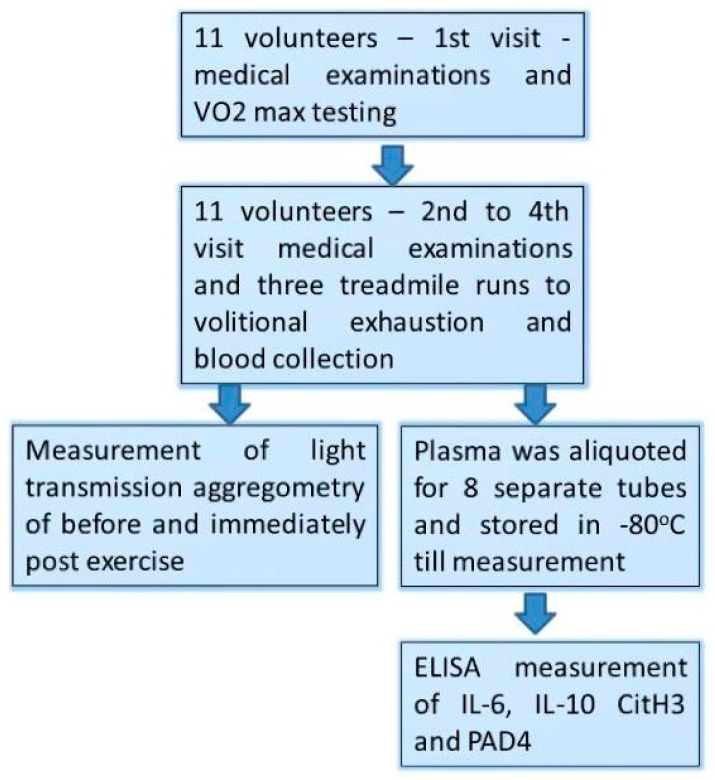
Study design flow chart.

The study design was the same as in our previous reports [11,16]. Figure 1 shows point by point:

(1) Those male volunteers who fulfilled the inclusion/exclusion criteria underwent a treadmill VO_2_max test at the first visit (day 1st).
(2) At the three consecutive visits separated by 72 h of resting period (day 7th, 10th, and 13th), participants performed a treadmill exercise to exhaustion at speed matching to 70% of their personal VO_2_max.
(3) Venous blood (2.7 mL) was collected into vacutainer tubes with EDTA and sodium citrate (Becton Dickinson, Franklin Lakes, NJ, USA) before and just after each bout of exhaustive exercise.
(4) Light Transmission Aggregometry (LTA) was executed immediately after the blood collection, while the obtained plasma was aliquoted, frozen, and stored at −80 °C until the measurements.

### 2.3. Variables Measured with ELISA Test

Histones/PAD4 and interleukins were measured in EDTA plasma. Quantitative plasma levels of citrullinated histone H3 and PAD4 were measured using ELISA Kit purchased from Cayman Chemical (Ann Arbor, MI, USA), the assay range was 0.15–10 ng/mL. IL-6 and IL-10 were tested using Diaclone kit (Besançon, France) with kit sensitivity 2 pg/mL. Pre-exercise interleukin 6 levels remained below the detection limit below the detection limit of ELISA kit (2 pg/mL), thus half of the detection limit has been calculated (which corresponds to a normal level of this interleukin in healthy individuals (0.8–1.0) [23]. Samples were carried out according to the manufacture’s instruction. Readings were performed on 96 plate reader VICTOR X Multilabel Plate Reader (Perkin Elmer, Wellesley, MA, USA) at 450 nm. The linear interval was defined as the linear section of the best-fit standard curve. Each standard curve was fitted using a four-parameter logistic (4PL) regression, and the 95% confidence interval (95% CI) was considered.

### 2.4. Platelets Function Measurement

Platelet function testing was measured using LTA (Light Transmission Aggregometry) [24]. In brief, blood was collected on citric acid and then platelet aggregation was measured photometrically using a Chronolog 700 Aggregometer (Chronolog Corp., Havertown, PA, USA). Aggregation was induced by 1 μmol/L adenosine diphosphate (ADP) (Sigma-Aldrich, Vienna, Austria). The duration of platelet aggregation run ranged from 5 to 10 min. The intensity (A), lag (L), time (T), and the rate (V) of aggregation were determined from the aggregation plot.

### 2.5. Other Variables

The measurement of serum creatine kinase (CK), aspartate aminotransferase (AST), alanine aminotransferase (ALT), as well as concentrations of C-reactive protein (CRP), lactic acid, glucose, urea, and creatinine used for correlation analysis, have been described previously [11].

### 2.6. Statistical Analysis

Statistical analysis has shown that the estimated total sample size was calculated on the basis of an analysis of covariance (ANOVA) test with 5 categories. We assumed a significance level of 0.05, a power of approximately 0.95, a medium effect size 4 and sigma = 5. The desired sample sizewas 34 samples whereas we had in total 66 cases.

Results were expressed as a mean (SD) and median (interquartile range). Data distribution was tested with the Shapiro–Wilk’s W test. The analysis of variance was applied using ANOVA rang test for repeated observations followed by the Scheffe’s test or Friedman ANOVA followed by the post hoc Wilcoxon test. Detailed p-values have been shown in supplemental Table S6. The Spearman rank correlation (non-parametric test) was used to measure the degree of association between two variables. Statistical significance was set at *p* < 0.05. Statistical analysis was performed with the Statistica software v13.

## 3. Results

All included men successfully completed the protocol of three repeated exhaustive treadmill exercises. A significant increase in H3Cit and PAD4 was observed after each exercise session. The mean concentration of circulating citrullinated histones increased by 93.5, 67.9, and 23.5 percent in response to the first, second, and third bout of performed exercise (Table 1). While PAD4 increased 51.7, 84.8, and 36.55 percent in response to the first, second, and third bout, respectively. Pre-exercise H3Cit did not change during the study, although the analysis of variance for repeated measures showed that the decreasing trend of analyzed H3Cit is statistically significant (*p* = 0.000617). Individual results are shown in supplemental Tables S1–S5 and Figures S1–S2.

The mean level of Light Transmission Aggregometry (LTA) significantly increased by about 34.8, 74.3, and 43.5 percent in response to the first, second, and third bout of exhaustive exercise respectively (Table 2). However, each bout seems to be an independent event because no adaptation or accumulation has been observed. The individual results are shown in supplemental Tables S1–S5.

Both IL-6 and IL-10 raised consequently in response to each bout of exercises. Interleukin 6 increased 8.4-, 8.1-, and 7.3- times, respectively in the first, second and third bout of exercise, whereas the increase of interleukin 10 was_2.6-, 2.9-, and 2.9-times, respectively, in the first, second, and third exercises session, respectively (Table 3). Individual results are shown in supplemental Tables S1–S5.

A strong correlation r = 0.79 has been observed between CitH3 and PAD4. Moreover there was a positive correlation between Il-6 and IL-10, H3Cit, and LTA. Additional analysis of the previously collected data confirms the positive correlation of LTA with cf nDNA and lactate both with aggregation and citrullinated histone H3 and PAD4. It is worth noting that although CitH3 and cf nDNA have no confirmed correlation, there was a positive correlation between PAD4 and cf nDNA. All correlations were shown in Table 4.

## 4. Discussion

Many markers of strenuous exercise rise to the same extent as changes caused by trauma, sepsis, or cardiac arrest. The increase in various classical biochemical markers such as troponin, creatine kinase, or aminotransferases have been observed also in response to exercises [11]. Numerous research have shown that cf nDNA rises over a dozen times in stress caused by exercises, which is similar to the increase caused by traumatic incidents, spread infection, or shock [3,6,11,12]. During nuclear decondensation, released DNA is followed by histones. It is worth mentioning that extracellular histone exhibits many similarities with cf nDNA as a pathology marker of sepsis or cancer [5,11,22,25]. However, to the best of our knowledge, extracellular histones have not been studied in response to exercises. This fact is essential for understanding post exercises physiological well-being since histones exhibit high cytotoxicity, and the ability to cause multiple organ endothelial cell dysfunction and inflammatory response [1,2,3,4,5]. In the present study, all healthy individuals had a normal low level of pre-exercises H3Cit/PAD4, but each bout of exercise raised the level of these proteins [22]. However, the growth of CitH3 was smaller with each subsequent treadmill run and decreased from 93 to 23 percent between the first and third bout of exercise.

In our previous study, we have observed the exact opposite trend regarding cf nDNA (rose 12-fold in the first bout vs. 17 times in the third one). Furthermore, the increase in histones is still six to eight times lower than the increase in cf nDNA. Basing on our previously published results, we have studied the association of cf nDNA with extracellular histone, but no correlation has been found. This was surprising, but we assume that it might be due to different cellular releases (transport from the nucleus) or more probably dissimilar kinetics of degradation of these molecules. Both cf nDNA and circulating histones have very dynamic degradation kinetics. Though, cell-free nDNA is degraded mostly by serum DNase activity, while extracellular histones are caught by kidneys or liver [26]. Both are cleared from circulation within minutes after release. In our study, blood was collected immediately after cessation of exercise, so such significant differences should be caused by degradation or capture occurring during the period of exercise. Alternatively, histones might bind to plasma proteins or endothelial cells thus, consequently, being undetermined by ELISA antibodies.

Once cell-free nDNA or histones are released into the extracellular space, they can be called DAMPs (damage-associated molecular patterns). DAMPs, called also alarmins, are host biomolecules that can initiate and spread a non-infectious inflammatory response and might be partly responsible for negative side effects of physiological stress. Histones binding to the cell membranes induce Ca^2+^ influx into the cells causing harmful effects in adjacent cells [27]. Beither et al. reported that level of chromatin nonhistone protein, HMGB1 (High Mobility Group Box 1), increased in response to strenuous treadmill run 3.3 times, whereas cf nDNA increased 14 times. Moreover, in their study, the authors observed a positive correlation between these molecules [12]. On the other hand, twelve weeks of Nordic walking activity, combined with vitamin D supplementation, in a group of elderly women, decreased serum HMGB1. This suggests that regular exercise might diminish the alarmins response in healthy adults [17]. We have observed a significant trend of CitH3 between bouts which might confirm this phenomenon.

The presence of citrullinated histones seems to be the most reliable marker of NETosis [28]. Further observed repeated response of PAD4 might confirm association of NETosis with strenuous exercises. MPO (myeloperoxidase) concentration, which is another common marker of NETosis, rises in response to exercise and correlates with the amount of cf nDNA released [13]. It is noteworthy that, the lactate accumulation, characteristic for exercises, might impair the release of histones and NETs formation [29]. However, how leukocytes release histones (or cf nDNA) is still uncertain, it might be NETosis, spontaneous release, or less likely apoptosis or necrosis. However, apoptosis or necrosis takes even a few hours to release nuclear compartments; therefore, it seems be to too long to occur during a single bout of exercise.

Extracellular histones might function as microbicidal proteins by the pro-thrombotic activity, limiting the spread of infection or isolating areas of injury, which allows for immunological activity. However, H3Cit toxicity is not specific to pathogens and contributes as well to tissue damage, which, in cases of systemic inflammation, may lead to multiorgan failure and finally to death. Research of the processes of histone release in acute inflammation and the mechanisms of histone-related tissue toxicity allows to develop therapeutic strategies, for targeting histones in acute inflammatory diseases [1,2,3,4,22]. To this end, we sought to examine how significantly histones contribute to exercise-induced pathologies. Here, we observed that H3Cit increased slightly, compared to sepsis or trauma. In trauma, the median H3Cit level was 28.6 ug/mL vs. 2.3 ug/mL in healthy volunteers [1]. In sepsis, H3Cit was increased fourteen times for the whole seven day period [30,31]. Thus, in this context, a very low increment of H3Cit might suggest limited histone induced cytotoxicity caused by exercise.

Platelets play a fundamental role in normal hemostasis, while their acquired dysfunctions are involved in a variety of thrombotic events or cardiovascular disease (CVD) development [32]. Platelet activation can be triggered by several specific platelets stimulating mediators including ADP. In our study, we observed a significant increase in ADP induced platelet aggregation. Furthermore, the increase was repeatable in each bout of exercise, and presented no signs of tolerance or accumulation. Similarly, Tozzi-Ciancarelli et al. showed that a single bout of strenuous exercise to exhaustion induced a significant increase in evoked platelet aggregation, whereas exercises at moderate intensity decreased platelets sensitivity. Our study design included three treadmill runs to exhaustion and LTA increased similarly. Their research showed a rise from 38 to 65 percent, whereas in our study the increase of LTA was from 41 to 55 percent in the first bout [33]. We might speculate that, if a participant reaches a much higher distance, exercises have a lower load for him than for the others. A phenomenon in which moderate exercises decreases platelet activity in contrast to exhaustive ones seems to be confirmed by other studies [34,35]. To sum up, reports considering platelet aggregation induced by ADP and association with exercises are inconclusive. The results of some studies are in contrast to our results and suggest a decreased platelet activity or personal-dependent mechanism, which divides participants into responders and non-responders [35,36].

Neutrophil extracellular traps (NETs) are suspected to be an important link between inflammation and thrombosis. However, in our study, we did not observe any association between citrullinated histones/PAD4 and aggregation [28]. We might speculate that either histones interact insufficiently or rise too low to trigger platelet aggregation [26]. The mechanism which explains the influence of physical activity on platelet responsiveness is probably very complex and involve many different processes such as free radicals, leucocyte activity, and metabolites including cf nDNA. In this context, circulating histones might not be fundamental. However, histones origin from histone-DNA complex and the effect should not be separated. Furthermore, a correlation has been observed when we combine them with our previously published results of cell free nDNA. Platelets activating effect might be shielded when cf nDNA is in complex with histone component [37]. It is worth emphasizing, when the positive charge of histone H3, and H4 was neutralized, then the induced aggregation was inhibited [38].

In the present study, experimental results indicated that exercise increased IL-6 and IL-10 to approximately 8 pg/mL and 6 pg/mL, respectively, and similarly in every accomplished bout. These data are analogous to those of other similar research [7]. The cytokine hypothesis considers that inadequate recovery induces musculoskeletal trauma, increasing the production and release of proinflammatory cytokines, mainly IL-6 [7]. The anti-inflammatory effect of physical exercise training can be mediated through the induction of an anti-inflammatory environment, such as IL-10 [39,40]. IL-6 is released by immune cells and after the stimulation of skeletal muscle fibers. This protease-dependent release of IL-6 might be initiated by lactate production, linking training intensity and lactate production to IL-6 release during strenuous exercise [11]. Moreover, epidemiological studies on healthy individuals reveal that significantly higher levels of IL-6 are associated with the risk of cardiovascular events [41]. Recently, Thalin et al. in cancer patients have reported that IL-6 positively correlated with H3Cit. In addition, in our study, we have observed that IL-6 was positively correlated not only with H3Cit but also with IL-10, and blood aggregation confirmed the proinflammatory association of these variables [42].

### Study Limitations

(1) Our study has several limitations, a relatively small number of subjects, and hence the inability to divide participants into subgroups (well-trained/untrained volunteers, female/male).
(2) Second, the lack of kinetic analysis makes it impossible to confirm the trend of citrullinated histones or explain the mechanism that disrupts the relationship between circulating histone and cfDNA.
(3) Finally, since CitH3 ELISA kit detects both DNA bound and free histones this may result in uncertainty in some observations.

## 5. Conclusions

Herein we have noticed:

(1) This is the first study showing that the level of circulating histone and PAD4 protein increases in exhaustive exercises. Moreover, the presence of circulating histones in post-exercise serum might confirm the increase of NETosis process during exercise.
(2) Blood aggregation status and interleukin expression increase in response to each bout of strenuous exercise.
(3) Despite the parallel increase, no significant correlation between citrullinated histone or blood aggregation was found. However, positive correlations of cf nDNA with blood aggregation, and lactate with blood aggregation, and lactate with citrullinated histone have been observed.
(4) Although each bout caused an increase in histones, all parameters normalized three days after the treadmill run. However, histones showed a downward trend in their increment.
(5) The increment of H3Cit is relatively low comparing to the other diseases what might suggest limited exercise induced histone cytotoxicity.

## Figures and Tables

**Table 1:** Level of H3Cit and PAD4 before and after each of three bouts of exhaustive treadmill exercise.

Parameter	Bouts of Exhaustive Treadmill Exercise					
	1st Bout		2nd Bout		3rd Bout	
	Before	After	Before	After	Before	After
H3Cit ng/mL	3.08 ± 2.9 (1.96 ± 2.9)	5.96 ± 3.0 * (4.63 ± 4.4)	3.65 ± 3.5 (2.23 ± 3.4)	6.37 ± 5.2 (4.29 ± 5.4)	3.86 ± 3.1 (2.57 ± 4.4) †	4.75 ± 3.04 * (5.05 ± 5.7)
PAD4 ng/mL	2.03 ± 3.2 (0.68 ± 0.6)	3.08 ± 3.7 * (1.40 ± 1.3)	1.91 ± 3.4 (0.41 ± 0.6)	3.53 ± 4.1 * (1.42 ± 1.8)	2.38 ± 3.6 (0.67 ± 1.6)	3.25 ± 3.8 * (1.67 ± 2.4)

Results are expressed as mean ± SD (median; IQR). * vs. corresponding value before the bout, *p* < 0.05.

**Table 2:** Level of LTA status before and after each of three bouts of exhaustive treadmill exercise.

Parameter	Bouts of Exhaustive Treadmill Exercise					
	1st Bout		2nd Bout		3rd Bout	
	Before	After	Before	After	Before	After
LTA U/min	40.8 ± 14.7 (37.2 ± 15.5)	55.0 ± 14.3 * (56.7 ± 11.7)	33.3 ± 16.8 (31.1 ± 24.6)	57.0 ± 14.7 * (63.1 ± 17.2)	39.3 ± 12.4 (35.3 ± 13.3)	56.43 ± 14.77 * (57.05 ± 7.68)

LTA- Light Transmission Aggregometry. Results are expressed as mean  ±  SD (median; IQR). * vs. corresponding value before the bout, *p* < 0.05.

**Table 3:** Level of IL-6 and IL-10 before and after each of three bouts of exhaustive treadmill exercise.

Parameter	Bouts of Exhaustive Treadmill Exercise					
	1st Bout		2nd Bout		3rd Bout	
	Before	After	Before	After	Before	After
Il-6 (pg/mL)	1 ± 0 (1 ± 0) †	8.44 ± 12.84 * (2.43 ± 6.27)	1 ± 0 (1 ± 0) †	8.12 ± 9.59 (2.49 ± 6.31)	1 ± 0 (1 ± 0) †	7.28 ± 7.54 * (3.01 ± 6.3)
IL-10 (pg/mL)	2.63 ± 1.25 (2.66 ± 1.2)	6.81 ± 6.27 * (4.18 ± 5.56)	2.34 ± 1.13 (2.23 ± 0.26)	6.99 ± 11.09 * (2.77 ± 1.62)	2.02 ± 1.04 (2.08 ± 1.51)	6.04 ± 6.59 * (2.37 ± 6.1)

Results are expressed as mean ± SD (median; IQR). * vs. corresponding value before the bout, *p* < 0.05. †—Pre-exercise interleukin 6 levels remained below the detection limit of ELISA kit (2 pg/mL), thus half of the detection limit has been calculated (which corresponds to a normal level of this interleukin in healthy individuals (0.8–1.0) [23].

**Table 4:** Spearman’s (ρ) correlations between selected analyzed parameters before and after three repeated bouts of exhaustive treadmill exercise.

Spearman Rang Correlation Variables	Correlation Coefficient (r) Spearman R	*p*-Value
H3Cit vs. PAD4	0.786	(below 0.000005)
IL-10 vs. IL-6	0.374	(*p* = 0.0021)
H3Cit vs. Aggregation	0.157	(ns)
H3Cit vs. IL-6	0.312	(*p* = 0.0078)
PAD4 vs. IL6	0.485	(*p* = 0.000036)
H3Cit vs. IL-10	0.207	(ns)
Aggregation vs. IL-6	0.409	(*p* = 0.00049)
Aggregation vs. IL-10	0.051	(ns)
H3Cit vs. cf nDNA *	0.224	(ns)
PAD4 vs. cf nDNA	0.348	(*p* = 0.0041)
PAD4 vs. Aggregation	0.487	(*p* = 0.000092)
Aggregation vs. cf nDNA *		
Aggregation vs. Lactate *	0.412	(*p* = 0.00058)
H3Cit vs. Lactate *	0.266	(*p* = 0.030)
PAD4 vs. Lactate *	0.369	(*p* = 0.0023)

Calculations were performed in absolute numbers. * This research is a continuation of the previously published articles [11,16] which allows us to study the correlation with any previously investigated and published data; ns—non significant.

## Data Availability

The data presented in this study are openly available at supplemental materials.

## Supplementary Materials

The following are available online at https://www.mdpi.com/2079-7737/10/3/181/s1. S1. This file comprises the tables with determined individual results (XLSX).

## Abbreviations

PAD4	peptidylarginine deiminase 4
nDNA	Nuclear Deoxyribonucleic acid
NETs	Neutrophil extracellular traps
H3Cit	Citrullinated histone H3
IL	Interleukin
LTA	Light Transmission Aggregometry
CVD	Cardiovascular disease
DAMPs	Damage-associated molecular patterns
HMGB1	High Mobility Group Box 1
MPO	Myeloperoxidase
ADP	Adenosine diphosphate
CK	Creatine kinase
AST	Aspartate aminotransferase
ALT	Alanine aminotransferase
CRP	C-reactive protein
